# Waiting times for elective treatments according to insurance status: A randomized empirical study in Germany

**DOI:** 10.1186/1475-9276-7-1

**Published:** 2008-01-09

**Authors:** Markus Lungen, Bjoern Stollenwerk, Philipp Messner, Karl W Lauterbach, Andreas Gerber

**Affiliations:** 1 Institute of Health Economics and Clinical Epidemiology, University of Cologne, Gleueler Str. 176 – 178, D-50935 Cologne, Germany; 2 Department of Public Health, Medical Decision Making, UMIT – University for Health Sciences, A-6060 Hall, Austria

## Abstract

**Background:**

Health insurance coverage for all citizens is often considered a requisite for reducing disparities in health care accessibility. In Germany, health insurees are covered either by statutory health insurance (SHI) or private health insurance (PHI). Due to a 20%–35% higher reimbursement of physicians for patients with PHI, it is often claimed that patients with SHI are faced with longer waiting times when it comes to obtaining outpatient appointments. There is little empirical evidence regarding outpatient waiting times for patients with different health insurance status in Germany.

**Methods:**

We called 189 specialist practices in the region of Cologne, Leverkusen, and Bonn. Practices were selected from publicly available telephone directories (Yellow Pages 2006/2007) for the specified region. Data were collected for all practices within each of five specialist fields. We requested an appointment for one of five different elective treatments (allergy test plus pulmonary function test, pupil dilation, gastroscopy, hearing test, MRT of the knee) by calling selected practices. The caller was randomly assigned the status of private or statutory health insuree. The total period of data collection amounted to 4.5 weeks in April and May 2006.

**Results:**

Between 41.7% and 100% of the practices called were included according to specialist field. We excluded practices that did not offer the requested treatment, were closed for more than one week, did not answer the call, did not offer fixed appointments ("open consultation hour") or did not accept any newly registered patients. Waiting time difference between private and statutory policyholders was 17.6 working days (SHI 26.0; PHI 8.4) for allergy test plus pulmonary function test; 17.0 (25.2; 8.2) for pupil dilation; 24.8 (36.7; 11.9) for gastroscopy; 4.6 (6.8; 2.2) for hearing test and 9.5 (14.1; 4.6) for the MRT of the knee. In relative terms, the difference in working days amounted to 3.08 (95%-KI: 1,88 bis 5,04) and proved significant.

**Conclusion:**

Even with comprehensive health insurance coverage for almost 100% of the population, Germany shows clear differences in access to care, with SHI patients waiting 3.08 times longer for an appointment than PHI patients. Wide-spread anecdotal reports of shorter waiting times for PHI patients were empirically supported. Discrepancies in access to care not only depend on accessibility to comprehensive health insurance cover, but also on the level of reimbursement for the physician. Higher reimbursements for the provider when it comes to comparable health problems and diagnostic treatments could lead to improved access to care. We conclude that incentives for adjusting access to care according to the necessity of treatment should be implemented.

## Background

A large number of studies have investigated and provided evidence of differential treatment of patients according to ethnic membership, sex and geographical region [1-4]. There are, however, few studies which have focused on the effects of insurance cover on the accessibility of health care services and the allocation of appointments for elective treatments and in turn upon reduced waiting times [5-7]. Studies in the US demonstrated longer waiting times for appointments following a reduction of Medicare reimbursement based on randomized telephone calls with randomly assigned insurance types [8,9]. It was shown that, in comparison to Medicare insurees, privately insured or out-of pocket-payers were more likely to receive an outpatient appointment within one week [10]. Within paediatric care, data from California have pointed to differences in treatment according to insurance status in the fields of surgery, urology, and orthopaedics [11-13]. There is evidence to suggest that physicians are responsive to financial incentives [14,15]. Patients' insurance status could be an indication of the level of financial reimbursement for the physician. Anecdotal reports in Germany generally conjecture that a patient's insurance status influences the allocation of appointments by registered physicians in private practices. To date, there are no studies in which physicians have been randomly contacted regarding appointment requests.

This issue is of particular relevance. While insurance coverage is available to almost all citizens in Germany, the type of insurance status has an effect on the amount which the physician is reimbursed. Approximately 10% of the German population is covered by comprehensive private health insurance. This form of insurance is not open to all segments of the population, but is compulsory for civil servants and optional for the self-employed and individuals with an annual income of more than 47.250 Euros (60.000 US$; 32.500 GBP). It is not possible for other population groups to take out a comprehensive private insurance policy, though supplementary private insurance packages remain an option. These additional policies currently only offer a modified range of services within in-patient care, including treatment by the chief consultant and allocation of a bed in a single room.

Given that private insurance premiums are based on morbidity and age and statutory comprehensive health insurance contributions on income, the extent to which insurance from a private company leads to increased policyholder contributions or premiums in individual cases can not be discerned. It is due to these premium guidelines that groups of individuals with lower morbidity and higher income tend to be more greatly represented within comprehensive private insurance.

A patient's status as private policyholder signifies a revenue for the physician which is approximately 20–35% higher than that which is to be gained in treating statutory insurees. For this reason it has been conjectured that physicians are more inclined to offer private policyholders an appointment and that ensuing treatment of these patients is carried out more efficiently. Given that out-patient care in Germany is almost entirely provided by private practices (and not by outpatient clinics/departments or hospital ambulatory care centres), potential consequences for the general health care situation are immense.

The following study investigate whether private patients are preferred over statutory health insurance policyholders in the allocation of appointments for elective outpatient treatments.

## Methods

An appointment was requested from those practices included in the current study by means of a telephone call. In Germany, requesting an appointment per telephone is a standard procedure when it comes to making initial contact regarding elective treatments. For each of the selected specialist fields, appointment requests were made using standardized conversation guidelines within a period of a few days. The total period of data collection amounted to 4.5 weeks in April and May 2006. In a randomized manner and with a probability of 50%, the caller claimed to be either privately or statutorily insured. Each practice was thus only called for one appointment. We assumed that repeatedly calling using the same conversation script could attract unnecessary attention. The caller did not request an appointment in the near future and further did not provide any exceptional reasons for the allocation of an appointment at either an earlier or later date. In the case that the particular health insurance company was asked for, a market leading company in the respective insurance sector was named. Upon inquiry of the name of the referring physician, a fictitious physician from a distant region was named and a recent move to the present area referred to.

### Selection of physicians' practices

Practices were selected from publicly available telephone directories (Yellow Pages 2006/2007) for the region Cologne/Bonn/Leverkusen. Data were collected for all practices listed under each specialist field in the directory. Given that the number of practices in the region of Cologne was below a threshold of 20, the selection was expanded to include the neighbouring regions of Leverkusen and Bonn. Criteria for the exclusion of practices were as follows: 1. Practice did not offer the treatment in question. 2. Practice was currently closed for more than one week of vacation. 3. Practice proved unattainable after three attempted calls at various points in time during the investigation (not including cases of "line busy"). 4. Practice offered only an open consultation hour and no fixed appointments. 5. Practice treated only private patients or did not accept newly registered patients. 6. Practice no longer existed. Each practice was randomly called by either a supposedly private or supposedly statutory health insuree. Insurance status was mentioned during each phone call.

### Selection of interventions and region

Interventions were selected according to the following criteria: 1) Treatment should not be considered an emergency, but rather an elective intervention. 2) Specialist care, however, was indicated. 3) Condition is relatively prevalent among the population. Interventions and specialist fields of the physicians are displayed in Table 1. In Germany, all five selected interventions are generally performed during a single elective appointment/visit and are often handled on the basis of an outpatient referral from a general practitioner to a specialist.

**Table 1:** Number of practices included in the study according to specialist field and intervention

Specialist field	Intervention	Data collection for all practices	Number of physicians' practices	Number of practices included
Allergology/Pulmonology	Allergy test plus pulmonary function test	Yes (Cologne/Bonn/Leverkusen)	SHI*:12	SHI: 5 41.7%
			PHI**: 12	PHI: 5 41.7%
Ophthalmology	Pupil dilation	Yes (Cologne)	SHI: 30	SHI: 22 73.3%
			PHI: 31	PHI: 22 71%
Gastroenterology	Gastroscopy	Yes (Cologne/Bonn/Leverkusen)	SHI: 6	SHI: 5 83.3%
			PHI: 5	PHI: 5 100%
Oto-rhino-laryngology (ENT)	Hearing test	Yes (Cologne)	SHI: 31	SHI: 22 71%
			PHI: 31	PHI: 24 77.4%
Diagnostic Radiology	MRT of the knee	Yes (Cologne/Bonn/Leverkusen)	SHI 16	SHI: 8 50%
			PHI 15	PHI: 10 66.7%

MRT = Magnetic resonance tomography

* SHI = statutory health insurance

** PHI = private health insurance

Requests for appointments for a colonoscopy (gastroenterology) and arthroscopy of the knee (orthopaedics) were also initially planned. These were, however, excluded on account of the emerging fact that colonoscopy patients must first take part in informational courses (colonoscopy as a preventative measure) and that arthroscopies are almost exclusively carried out in outpatient hospitals.

The region Cologne, Leverkusen and Bonn is homogeneous with regard to population density and the subsequent geographical distribution of physicians' practices and specialty practices. The region is typical of an urban West German setting.

### Statistical analyses

Both univariate analysis, referring to one specialist field at a time, and multivariate analysis, in terms of a multivariate regression model, were performed. For univariate analysis the Wilcoxon rank sum test, also known as Mann-Whitney test, was performed. Furthermore, the average of days of waiting time (working days) and the corresponding standard deviation were reported for each payment source.

For multivariate analysis, the waiting time (response variable) was modelled using a generalized linear model (GLM) from the quasi-Poisson-family with the log link function [16,17]. The number of working days between phone call and appointment was employed as response variable. Health insurance status (indicator variable) and specialist field (factor variable) were introduced as regressor variables. The interaction between specialist field and insurance status was not included in the model equation, as this effect did not reach significance (p = 0.40). The 5% significance level was used for all statistical tests.

## Results

A total of 189 practices were called; the inclusion rate varied between 41.7% and 100% depending on specialist field and health insurance status (Table 1). A total of 61 of the 189 practices were excluded in line with the criteria listed above. The number of practices excluded is displayed in Table 2 according to each of the six criteria. Most practices were excluded because of the lack of offering the service or treatment chosen for the specialty (34.4% of all exclusions). Only limited relevance had the offering of open consultation hours (16.4%) which might lead to a first-come first-serve line with no preferential treatment of PHI patients. However, anecdotal evidence indicates that open consultation hours lead to an even more considerable reduction in waiting times for PHI patients.

**Table 2:** Reasons for exclusion of practices initially selected from the telephone directory

Reason for exclusion	Total number of practices excluded n = 61)	Percent of excluded practices (61 = 100%))
Service/Treatment in question not offered, e.g. pulmonary function test	21	34.4
Practice closed for vacation for more than 1 week after the initial call (as indicated on the answering machine)	6	9.8
Practice not attainable after three attempts (excluding "line busy")	4	6.6
Practice offering only open consultation hours	10	16,4
Exclusive service for private insurees	13	21.3
Practice no longer in service	7	11.5

The number of included offices was highest in the specialist field ENT (46 included offices) and lowest in the specialist fields "Allergology/Pneumology" and "Gastroenterology" (10 included offices each).

Waiting time differed significantly between public and private insured in all but one specialist field (see Table 3). The only exception was the specialist field "Allergology/Pneumology" (p = 0.207). The relative difference was lowest in the specialist field "Allergology/Pneumology", where public insured had to wait 1.4 times as long and highest in "Diagnostic Radiology", where public insured had to wait 16.8 times longer than private insured. However, the relative differences in waiting time between the specialist fields was not significant (p = 0.40). The overall relative difference, as derived from the multivariate regression model, amounted 3.1 (95% confidence interval: [1.9–5.0]).

**Table 3:** Test statistics

Specialist field	Public insurance			Private insurance			Test statistic W	p value	Ratio
				
	Mean	Std.	N	Mean	Std.	N			
Allergology/Pneumology	19.8	6.3	5	14.6	6.2	5	6.0	0.207	1.4
Ophthalmology	24.9	32.7	22	8.5	13.9	22	128.5	0.008	2.9
Diagnostic Radiology	18.5	17.0	8	1.1	1.7	10	0.5	<0.001	16.8
Gastroenterology	40.4	28.7	5	8.2	6.1	5	1.0	0.020	4.9
ENT	6.0	6.7	22	2.9	5.3	24	168.0	0.031	2.1

Std. = standard deviation; N = number of subjects

The parameter estimates of the multivariate regression model are presented in Table 4. No parameter estimates are presented for the insurance status SHI and for the specialist field "Allergology/Pulmonology", because they serve as reference.

**Table 4:** Parametric coefficients

	Estimate	Std. Error	exp(Estimate)	95% CI	t value	Pr(>|t|)
(Intercept)	3.257	0.329	n.r.	n.r.	9.908	<0,001***
Private insurance policyholder	-1.124	0.251	0.32	[0.20, 0.53]	-4.475	<0,001***
Specialist field Ophthalmology	-0.032	0.359	0.97	[0.48, 1.96]	-0.089	0.929
Specialist field Diagnostic Radiology	-0.608	0.466	0.54	[0.22, 1.36]	-1.305	0.194
Specialist field Gastroenterology	0.346	0.422	1.41	[0.62, 3.23]	0.819	0.414
Specialist field ENT	-1.348	0.440	0.25	[0.11, 0.62]	-3.064	0.003**

Response: Waiting time (days); link-funktion: logarithm; family: quasi-Poisson; model type: generalized linear model; n.r. = "not relevant")

Significance levels of the regressor variables "Health Insurance Sstatus" and "Specialist Field" are presented in Table 5. Both variables had a significant effect on waiting time: "Health Insurance Status" (p < 0.001) and "Specialist Field" (p = 0.005).

**Table 5:** Significance of model terms

	Df	F	p-value
Insurance status	1	20.029	<0,001
Specialist field	4	5.453	0.005

Response: Waiting time (days); link-funktion: logarithm; family: quasi-Poisson; model type: generalized linear model.

The expected number of days to be waited leading up to appointment date, according to the multivariate regression model, is presented in Table 6 for each of the interventions according to insurance status. The absolute difference in waiting times between SHI and PHI patients is highest in "Gastroscopy" (24.8 days; SHI: 36.7; PHI: 11.9) and lowest in "ENT" (4.6 days; SHI 6.8 days; PHI 2.2).

**Table 6:** Number of working days to be waited leading up to appointment date according to insurance status and specialist field/intervention

	Working days to be waited leading up to intervention			
Intervention/Examination	SHI	PHI	Difference in working days	Difference expressed in % of PHI-waiting time

Allergy test and pulmonary function test	26.0	8.4	17.6	210%
Pupil dilation	25.2	8.2	17	207.3%
MRT of the knee	14.1	4.6	9.5	206.5%
Gastroscopy	36.7	11.9	24.8	208.4%
Hearing test	6.8	2.2	4.6	209%

## Discussion

Using a selection of elective out-patient examinations/interventions in five specialist fields, we investigated the extent to which insurance status influences the allocation of next possible appointments. Earlier appointments were offered to privately insured patients by physicians' practices in all specialist fields. In terms of relative differences, publicly insured had to wait about three times longer than privately insured.

Resneck et al. (2004), who conducted a similar study based on randomly assigned insurance types, also found a strong impact of reimbursement level on appointment waiting times in the field of dermatology. [8] While the length of waiting times was generally higher than in Germany (30 to 50 days), the study revealed a potential influence of regional differences in market shares and level of reimbursement. Furthermore, all previous studies using telephone calls to request a hypothetical appointment have shown that reimbursement levels have a strong impact on waiting times [7-10,18]. Hu and Reuben (2002) have, however, pointed out that the structure of managed care in the health care market rather than reimbursement per se, could influence practice patterns in terms of the time spent with the patient and hence access to care [19].

Several limitations of our trial should be noted. Firstly, it was performed in only one geographical region, without testing whether these findings are also representative of other areas. The region observed in this study features a rather high percentage of private insurance policyholders (as typical of cities). Whether this results in potentially longer or shorter waiting times for those with private insurance currently remains unclear. On the one hand, it is possible that a higher percentage of privately insured individuals may lead to less competition between practices in gaining private patients, which in turn could entail longer waiting times for this group of patients. On the other hand, a high percentage of PHI policyholders may permit more practices which exclusively serve this sector, so that waiting times for these patients is even shorter. As can be seen in Table 2, 13 of the 189 practices selected from the telephone directory were excluded due to the fact that they only accepted private insurees; this is equal to 7% of the total practice sample. Since these practices were excluded, it remains unclear whether waiting time differences between SHI and PHI insurees are even larger when these practices are included. Nevertheless, our finding clearly underscores that discrepancies in physicians' reimbursements are associated with disparities in access to treatment.

Secondly, the selection of elective interventions in the present study included only those which are not known to be associated with inferior quality of medical results for patients with later appointments. It is, however, largely refuted that a relationship between quality of results and waiting time does not exist. Prentice and Pizer (2007) found evidence of an association between long waiting times for outpatient health care and negative health outcomes such as mortality for patients of Veterans Affairs medical centres [20]. At the very least, the perceptions of the patient, together with his/her uncertainty regarding the expected treatment or examination results, appear to result in a worsening of the subjective situation.

## Conclusion

It is our view that the findings of the current study carry considerable consequences for health policy. According to these findings, the introduction of comprehensive health insurance coverage is not adequate in establishing equal access to medical treatment for all citizens. Rather, possible discrepancies in the level of reimbursement between individual insurance status' have an immense influence on access to health services in the case of identical illnesses.

In Germany, a stringent distinction between private and statutory health insurance prevails, which does not apply to additional, but rather to comprehensive insurances. Individuals with a higher income and lower morbidity tend to have improved access to medical services through comprehensive private insurance. It is in part argued that unequal access is to be found in all health systems, in so far as differences in income, willingness-to-pay and correspondingly susceptible and amenable physicians exist. This is no doubt true. However, private insurance in Germany is clearly restricted to population groups with evident risk selection and financial favouritism. A lively debate is currently underway in Germany concerning the harmonization of reimbursement modi for SHI and PHI patients. The alternative of introducing regulatory guidelines or even legal regulations for the safeguarding of equal access to care for all insurees is not applicable to the German situation. Firstly, it is obvious that a guideline would never be successful in forcing physicians to rearrange a waiting list, when strong financial incentives cause them to prefer certain groups of patients. Soft factors that are hard to control for, such as for example clinical conditions, would always lead to advantages for those patient groups that warrant higher reimbursement. Secondly, unlike other countries, there are no combined reimbursement schemes in Germany. Indeed, only two schemes exist: one for SHI insurees and the other for PHI insurees. The schemes mainly differ in the add-on factor found in the reimbursement of treatment provided to PHI insurees. Thirdly, both reimbursement schemes in Germany closely resemble a legal guideline. The PHI-scheme has the status of a government action, while the SHI-scheme is negotiated between the Federal Association of Physicians and health insurance companies. Harmonising reimbursements for the treatment of SHI and PHI patients in Germany is more a political than a legal issue.

In summary, our study demonstrates that reimbursement models have an impact on patient access to treatment, a fact often previously denied in Germany. From a health policy perspective, it is to be concluded that insurance coverage for the total population is not adequate in ensuring equal access to medical services. Rather, insurance-dependent reimbursement also influences the access possibilities of the patient. In aspiring for equality, an assimilation of reimbursement models or the possibility of an effortless change between insurance systems would be recommendable. Access to care should be adjusted according to the necessity of treatment.

## Competing interests

ML, BS, KWL and AG have received funding from statutory health insurance funds for various research projects in the last 5 years.

## Authors' contributions

The study was designed by ML, AG and KWL and drafted in written form by ML. Statistical analyses were conducted by BS. Physicians were contacted (via telephone) by PM who was also responsible for data management. All authors read and approved the final manuscript.
